# 

**Published:** 1893-05-20

**Authors:** 


					PPIOC TU/AnPUAr WW ^=-~ - WITH EXTRA NURSING SUPPLEMENT
EE'S? TWOPENCE. (j^tr^ It# HM *er Anxmni.8..e*..poat free.
W? Vol. XIV. May 20th, 1893. ^\1 JJ% Monthly Part*. 6d.i post free, 84.
bSJS2?.'! ^ Qenaral Foot Office m a Newspaper far J^b ^^5^ Ditto per Aunmi 8s., post free.
o?ais?on in the United Kingdom and Abroad.] ^
No. 347. Vol. XIV.] 8ATT7BDAT,
May 20th, 1893.
[Twopence.

				

